# Socioeconomic inequalities in intergenerational overweight and obesity transmission from mothers to offsprings in South Africa

**DOI:** 10.1016/j.ssmph.2022.101170

**Published:** 2022-08-06

**Authors:** Mweete D. Nglazi, John E. Ataguba

**Affiliations:** a Health Economics Unit, School of Public Health & Family Medicine, University of Cape Town, Cape Town, South Africa; b Department of Community Health Sciences, Max Rady College of Medicine, Rady Faculty of Health Sciences, University of Manitoba, Winnipeg, Canada

**Keywords:** Intergenerational transmission, Socioeconomic inequality, Overweight, Obesity, South Africa

## Abstract

This study assesses socioeconomic inequality in the intergenerational transmission of overweight and obesity from mothers to offsprings in South Africa, including the factors contributing to inequality. Data were drawn from the 2017 National Income Dynamic Study, which collected anthropometric and socioeconomic information. Non-pregnant mothers aged 15–49 years and their offsprings 0–14 years were included in the analysis. The dependent variables used in the study were the intergenerational transmission of overweight and obesity. Socioeconomic inequality was assessed using the concentration index. A positive index means that intergenerational overweight and obesity is more likely among the wealthier populations, while a negative index signifies the opposite. The concentration index was decomposed to understand the factors that explain inequalities in the transmission of overweight and obesity from mothers to offsprings. Concentration indices for the intergenerational transmission of overweight and obesity were positive for boys (0.17) and girls (0.23). Thus the intergenerational transmission of overweight and obesity occurs more among wealthier mothers. Although factors explaining socioeconomic inequality in the intergenerational transmission of overweight and obesity differed by offspring sex, mother's marital status (+38%) and socioeconomic status (around +8%) were central determinants of socioeconomic inequalities in intergenerational overweight, while mother's smoking (around +25%), education (about +13%) and employment status (around +12%) contributed to intergenerational obesity inequality. Policies to reduce overweight and obesity burdens and the intergenerational transmission of overweight and obesity in South Africa should target women who bear a significant burden of overweight and obesity and could transmit them to their offsprings. The policies should also recognise the key factors explaining these socioeconomic inequalities. This approach will reduce the future burden of diseases associated with overweight and obesity in South Africa and improve the country's overall health outcomes.

## Highlights

• Intergenerational overweight and obesity occurs more among richer mothers.
• Mother's marital status influenced intergenerational overweight inequality.
• Intergenerational obesity inequality was attributed to mother's smoking status.
• Factors explaining intergenerational health inequalities differed by offspring sex.

## 1 Introduction

Overweight and obesity are associated with elevated risks for certain non-communicable diseases like cardiovascular disease, diabetes, and cancers (Flegal et al., 2013; Forouzanfar et al., 2015; Institute for Health Metrics and Evaluation, 2019; Ng et al., 2014; World Health Organization, 2019). In addition to the negative impacts of overweight and obesity on individuals, there is evidence of the intergenerational transmission of body mass index (BMI) and overweight or obesity from parents to offsprings (Anderson et al., 2007; Brown & Roberts, 2013; Classen, 2010; Classen & Hokayem, 2005; Classen & Thompson, 2016; Dolton & Xiao, 2015, 2017; Martin, 2008; Whitaker, 2004). This transmission is a complex process involving many factors such as genetics, cultural and environmental factors shared by parents and offsprings (Classen & Thompson, 2016; World Health Organization, 2007). The intergenerational transfer of overweight and obesity impacts health and may pose a substantial economic burden for both generations, reducing economic productivity and putting social protection systems under severe strain (Classen & Thompson, 2016; Ng et al., 2014; Republic of South Africa, 2015; Swinburn et al., 2011).

Despite evidence from South Africa and elsewhere on the intergenerational transmission of obesity (Anderson et al., 2007; Brown & Roberts, 2013; Classen & Hokayem, 2005; Classen, 2010; Classen & Thompson, 2016; Dolton & Xiao, 2015, 2017; Martin, 2008; Whitaker, 2004), few studies have examined the relationship between socioeconomic status and overweight/obesity across generations (Balasooriya et al., 2021; Zhang et al., 2011). Using the intergenerational mobility index based on the concentration index, a United States study by Zhang et al. (2011) measured the changes in socioeconomic inequality in obesity across generations and performed a decomposition analysis of the intergenerational mobility index to identify the factors that contribute to changing socioeconomic disparity in obesity. The decomposition analysis captured the effects of both changes in income distributions and changes in socioeconomic disparity in obesity simultaneously (Zhang et al., 2011). The study found that the intergenerational disparity in obesity across socioeconomic groups between fathers and their adult sons had reduced (Zhang et al., 2011). They also found a similar reduction in intergenerational disparity in obesity across socioeconomic groups between mothers and their adult daughters (Zhang et al., 2011). The decompositions suggested that changes in income distributions contribute smaller effects to changes in socioeconomic inequality in obesity between mothers and their adult daughters than between fathers and their adult sons (Zhang et al., 2011). Using regression-based decomposition on data from three waves of the Household Income and Labour Dynamics survey in Australia (2007 through 2013), Balasooriya et al. (2021) examined the effects of inherited contributing factors on body weight in adult children. They found demographic factors such as age, marital status, employment status, and living area to explain 4% of the inequality for BMI and overweight. Other circumstances like parental socioeconomic status accounted for between 20% and 25% of BMI and overweight inequalities (Balasooriya et al., 2021).

Intergenerational transmission of overweight and obesity could perpetuate existing socioeconomic inequalities between generations. This makes it essential to estimate and understand the factors contributing to the socioeconomic inequality in the intergenerational transmission of overweight and obesity, especially in countries with a significant proportion of adults living with obesity. In South Africa, 16% of adult men and 41% of adult women were living with obesity in 2016 (Non-Communicable Disease Risk Factor Collaboration – Africa Working Group, 2017). According to the NCD Risk Factor Collaboration estimates, South Africa had a global ranking (i.e., % obesity by country) of 112 in terms of adult male obesity and 23 in terms of adult female obesity in 2016 (Non-Communicable Disease Risk Factor Collaboration – Africa Working Group, 2017). The prevalence of adult female obesity is among the highest, especially in Africa. Moreover, the evidence is lacking concerning the socioeconomic inequality in the intergenerational transmission of overweight and obesity in South Africa. Therefore, this study aims to estimate and decompose the socioeconomic inequality in the intergenerational transmission of overweight and obesity from mothers to offsprings. Separate analyses were carried out for mother-son and mother-daughter pairs because intergenerational health transmission occurs across gender-specific lines (Pembrey et al., 2006). The evidence from this paper will be useful in South Africa, a country with a relatively high burden of obesity.

## 2 Material and methods

### 2.1 Data source and participants

This paper uses data from the 2017 wave of the National Income Dynamic Study (NIDS). The NIDS is a nationally representative longitudinal panel survey commenced in 2008 and repeated every two years. It is conducted by the Southern African Labour and Development Research Unit at the University of Cape Town and funded by the South Africa Presidency. Fieldwork for the 2017 wave was between February and December 2017 (Brophy et al., 2018). A total of 28,963 adults were interviewed in 2017 (wave 5), with a response rate of 68.6% and 96.2% at the household and individual levels, respectively. The overall survey response rate (householdresponserate×individualresponserates×BMIitemresponserate) was 53.3%. Details of the sampling procedure, including the sampling weights, are described elsewhere (Brophy et al., 2018). Briefly, the NIDS uses a stratified two-stage cluster sampling strategy to sample households at baseline. A total of 400 primary sampling units (PSUs) were selected in the first stage from Statistics South Africa's 3000 PSUs in the 2003 Master sample. In 2008, 7305 households were interviewed in 400 PSUs. All household members became a Continuing Sample Member (CSM) to be interviewed every two years. Children born to CSM women after Wave 1 are ‘born into’ the sample. Everyone currently living with a CSM (i.e., individuals referred to as Temporary Sampling Members [TSMs]) is also interviewed. As CSMs move out and start their households, the number of interviewees grows. A Top-Up CSM sample was introduced in Wave 5 to maintain national representativity. Note that the Top-Up CSM exists only in wave 5 and not in any preceding waves (including the original wave). Trained fieldworkers collected the data through standardised questionnaires; household questionnaire, adult questionnaire for adults aged 15 years and older, proxy questionnaire for non-available adults, and child questionnaires for children aged between 0 and 14 years. This paper used data from the adult (containing women of childbearing age), household and child questionnaires. First, a dataset of potential mothers containing variables derived from the adult and household questionnaire was created. Then, the mothers' dataset was linked to the child dataset using the mothers' identifier variable. There were 12,157 non-pregnant mothers aged between 15 and 49 years and 15,014 offsprings in the 2017 dataset. Only 10,735 matched pairs (mother-offspring) were retained, primarily due to missing information on the biological mothers of some children. From this number, 3329 paired observations had missing BMI-for-age and weight-for-height z-scores for offspring, leaving 7406 matched mother-offspring pairs in the final dataset for analysis. To note, there were no differences in the descriptive characteristics between the initial sample of 10,735 and the final sample of 7,406, suggesting that the missing BMI-for-age and weight-for-height scores were missing at random.

### 2.2 Definition of key variables

**Fig. 1:**
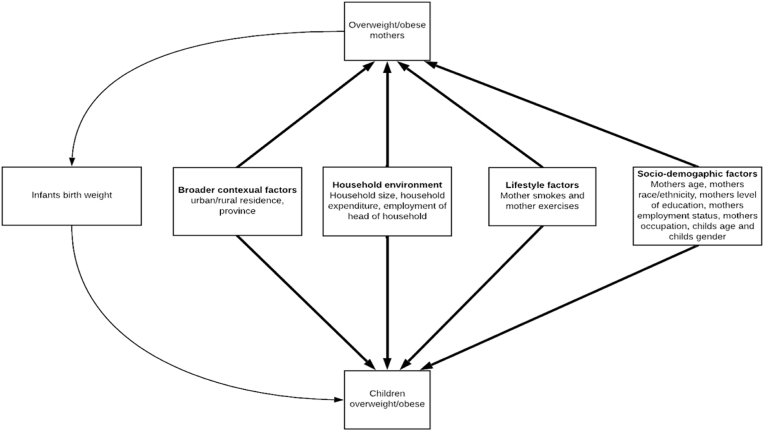
The intergenerational transmission of overweight and obesity.

Source: Adapted from World Health Organization (2007)

Table 1 contains a description of the key variables used in this paper. The variable selection was based on the model shown in Fig. 1, adapted from the WHO Regional Office for Europe (World Health Organization, 2007), data availability in the NIDS dataset and previous studies (e.g., Balasooriya et al., 2021). As shown in Fig. 1, overweight/obesity transfers from mothers to their children because heavier mothers, once they fall pregnant, tend to give birth to bigger babies that, in turn, have the propensity to be heavier children (World Health Organization, 2007). Although it is complex (Classen & Thompson, 2016), determinants such as socio-demographic characteristics, lifestyle, the household environment and broader contextual factors play a part in the intergenerational cycle of overweight and obesity (World Health Organization, 2007).

**Table 1:** A description of key variables used in the analysis.

Variable	Definition
Child's age	A child's age in years
Child overweight	Children under five years with a weight-for-height z-score of two or more standard deviations above the WHO Child Growth Standards median. Children 5–14 years with a BMI-for-age z-score of one or more standard deviations above the WHO Growth Reference median (World Health Organization, 2017). This variable was objectively measured.
Child obesity	Children under five years with a weight-for-height z-score of three or more standard deviations above the WHO Child Growth Standards median. Children 5–14 years with a BMI-for-age z-score of two or more standard deviations above the WHO Growth Reference median (World Health Organization, 2017). This variable was objectively measured.
Maternal overweight	A BMI ≥25 kg/m^2^ (World Health Organization, 2018). This variable was objectively measured.
Maternal obesity	A BMI ≥30 kg/m^2^ (World Health Organization, 2018)
Mother's age	A woman's age in years. This variable was objectively measured.
Mother's household size	The number of persons in a household
Socioeconomic status	Per capita household expenditure
**Mother's employment status**	
Employed	A woman who are in formal or informal employment
Unemployed	A a woman who are not in formal or informal employment (Reference category)
**Mother's population group**	
Black African^a^	Women self-identified as black African race (Reference category)
Coloured	Women self-identified as coloured
Indian/Asian	Women self-identified as Indian/Asian race
White	Women self-identified as white
**Mother's education**	
No schooling/	A woman with no education
primary education	A woman only primary education
Secondary	A woman with secondary education
Tertiary education	A woman with tertiary education
Vocational	A woman with vocational education
**Area of residence**	
Rural	A woman residing in a rural location (Reference category)
Urban	A woman residing in an urban location
**Mother's marital status**	
Married	A woman who is married (Reference category)
Living with partner	A woman who is living with a partner
Widow	A woman who is a widow
Divorced or separated	A woman who is divorced or separated
Never married	A woman who never married
**Mother smoking**	
Not smoking	A woman who reported not currently smoking
Smoking	A woman who reported currently smoking
**Exercise Habits**	
Never	A woman who never exercises (Reference category)
Less than once time a week	A woman who exercises less than once a week
Once a week	A woman who exercises once a week
Twice a week	A woman who exercises twice a week
Three or more times a week	A woman exercises three or more times a week
Quintiles of socioeconomic status (Quintiles 1–5)^b^	Quintile 1 = A woman is in the poorest socioeconomic group
	Quintile 2 = A woman is in the second poorest socioeconomic group
	Quintile 3 = A woman is in the middle socioeconomic group
	Quintile 4 = A woman is in the second richest socioeconomic group
	Quintile 5 = A woman is in the richest socioeconomic group
Intergenerational transmission of overweight from mothers to offsprings	1 = If both a woman and her offspring are living with overweight and 0 if this is not the case.
Intergenerational transmission of obesity from mothers to offsprings	1 = If both a woman and her offspring are living with obesity and 0 if this is not the case.

*Notes*.

a The South African population is predominantly black and racial disparities have been reported for obesity and overweight (Averett et al., 2014).

b Quintiles of socioeconomic status are based on household expenditure per capita.

#### 2.2.1 Maternal and offspring overweight and obesity

Maternal and offspring overweight and obesity status was based on objectively measured height and weight.

#### 2.2.2 Socioeconomic status

Per capita household expenditure was used to assess socioeconomic status (SES) in this paper. Socioeconomic quintiles were also generated using per capita household expenditure.

### 2.3 Analytical methods of estimating health inequality

Descriptive statistics were used to summarise data, with means for continuous variables and proportions for categorical variables. All estimates were adjusted for sampling weights. Offspring sex-stratified analyses were performed separately for the data.

### 2.4 Concentration index

The concentration index (Kakwani et al., 1997; Wagstaff et al., 1991) was used to measure or assess socioeconomic inequality in the intergenerational transmission of overweight and obesity from mothers to offsprings, with intergenerational transmission assessed using a dummy variable for having both a woman and her offspring living with overweight or obesity (see Table 1 for the description).

For simplicity and convenience, the standard concentration index (β) is computed as (Kakwani et al., 1997):

(1) $$2\sigma_{r}^{2}\left(\frac{h_{i}}{\mu }\right)=\alpha +\beta r_{i}+\varepsilon_{i}$$

where $\sigma_{r}^{2}$ is the variance of the fractional rank of socioeconomic status measure (r), α is the intercept, μ is the mean of the variable for the intergenerational transmission of overweight or obesity ($h_{i}$), and $\varepsilon_{i}$ is the error term (Kakwani et al., 1997; O’ Donnell et al., 2008). Traditionally, it was recommended to normalise the concentration index when a dummy variable is used. However, this normalisation can be problematic as it may produce counter-intuitive results (Ataguba, 2022). Thus, the analysis in this paper was based on the standard concentration index (β). A positive index (i.e., β>0) means that intergenerational overweight and obesity is more likely among richer than poorer women. Stated differently, overweight and obesity are more common among wealthier mother-offspring pairs than poorer mother-offspring pairs. A negative concentration index (i.e., β<0) signifies the opposite.

### 2.5 Decomposing the concentration index

The concentration index in Equation (1) was decomposed using the methods outlined in Wagstaff et al. (2003) to understand the drivers (i.e., contributions of the determinants) or factors that explain socioeconomic inequalities in the intergenerational transmission of overweight and obesity from mothers to offsprings. The contributing factors included in the decomposition analysis included household size, socioeconomic status, urban residence, child's age, mother's age, employment status, population group (race), education, marital status, smoking status, and exercise habits.

A positive value for the contribution of any factor means that it contributes to the concentration of intergenerational overweight or obesity among wealthier population groups than poorer groups. The opposite is the case for a negative value.

Consider an ordinary least squares regression model in Equation (2) that links the intergenerational transmission of overweight or obesity (i.e. the outcome denoted h) to a set of k determinants ($x_{k}$) where γ is the estimated coefficient on the determining factors.

(2) $$h_{i}=\alpha +\sum_{k}\gamma_{k}x_{ki}+\varepsilon_{i}$$

The concentration index for h, (i.e. C in Equation (3) but defined as β in Equation (1)) can be decomposed as:

(3) $$C=\sum_{k}\underset{deterministic}{\underbrace{\left(\gamma_{k}{\bar{x}}_{k_{i}}/\mu \right)C_{k}}}+\underset{unexplained}{\underbrace{GC_{\varepsilon }/\mu }}$$

where, μ is the mean of the outcome,h (i.e., intergenerational transmission of overweight and obesity from mothers to offsprings), $\bar{x}$ is the mean of determining factor or variable (k), $\gamma_{k}$ is the coefficient for each of the determinants in Equation (2), $GC_{\varepsilon }$ is the generalised concentration index for the error term (ε) (Wagstaff et al., 2003).

Equation (3) comprises two parts. The first part, the deterministic component, is the product of the elasticity of h with respect to each determining factor, denoted as $\left(\gamma_{k}{\bar{x}}_{k_{i}}/\mu \right)$ and the concentration index of the determining factor ($C_{k}$). This deterministic component is interpreted as the contribution of the determining factor (x) to socioeconomic inequality in the intergenerational transmission of overweight or obesity. The unexplained component is the generalised concentration index of the error term (ε) and should be close to zero in a well-specified model where all determining factors have been included in Equation (2) (Wagstaff et al., 2003).

## 3 Results

### 3.1 Description of the sample

As shown in Table 2, the mean age for offsprings (girls and boys) was estimated at 6.5 years (standard deviation (SD) 4.0 years), while the mean age for mothers was 32.8 ± SD 7.0 years. The mean household size was 5.8 ± SD 3.7 people. Overweight prevalence was higher for girls than boys, while obesity prevalence was similar for girls and boys. Nearly two-thirds of mothers were employed. Mothers had an overweight and obesity prevalence of 66.1% and 42.5%, respectively. Mothers were predominantly Black African, had attained secondary or higher levels of education, did not currently smoke and never exercised. Just over half of the mothers were never married. Nearly three-fifths lived in urban locations.

**Table 2:** Descriptive statistics for mothers aged 15–49 years and their offsprings in South Africa, 2017

	Mothers	Sons	Daughters	Both^a^
**Sample**	7406	3651	3755	7406
**Child's age years mean (SD)**	–	6.5 (3.94)	6.5 (4.00)	6.5 (3.97)
**Child overweight**	–	17.4 (15.4–19.6)	19.1 (17.3–21.0)	18.3 (16.9–19.7)
**Child obesity**	–	6.0 (4.6–7.6)	5.8 (4.8–7.0)	5.9 (5.0–6.9)
**Mother's age, years mean (SD)**	32.8 (7.03)	–	–	
**Household size**	5.8 (3.37)	–	–	–
**Mother's employment status**		–	–	–
Employed	64.7 (63.0–66.4)	–	–	–
Unemployed	33.5 (31.8–35.2)	–	–	–
Missing	1.8 (1.4–2.3)	–	–	–
**Mother's population group**		–	–	
Black African	86.4 (85.1–87.6)	–	–	
Coloured	8.3 (7.3–9.4)	–	–	
Asian/Indian	1.6 (1.2–2.1)	–	–	
White	3.7 (3.1–4.5)	–	–	
**Mother's education**		–	–	–
No schooling	1.2 (0.9–1.5)	–	–	–
Primary	6.4 (6.2–6.6)	–	–	–
Secondary	64.1 (62.4–65.8)	–	–	–
Tertiary	26.9 (25.3–28.7)	–	–	–
Vocational	0.3 (0.2–0.4)			
Missing	0.7 (0.5–1.0)	–	–	–
**Mother's marital status**		–	–	–
Married	32.3 (30.7–34.1)	–	–	–
Living with partner	9.5 (8.5–10.7)	–	–	–
Widow	2.7 (2.1–3.4)	–	–	–
Divorced or separated	2.8 (2.3–3.5)	–	–	–
Never married	51.7 (49.9–52.6)	–	–	–
Missing	1.3 (1.0–1.7)	–	–	–
**Area of residence**		–	–	–
Rural	38.5 (36.8–40.1)	–	–	–
Urban	60.4 (58.8–62.1)	–	–	–
Missing	1.1 (0.8–1.5)	–	–	–
**Mother smoking**		–	–	–
No	87.9 (86.7–89.1)	–	–	–
Yes	6.5 (5.7–7.5)	–	–	–
Missing	5.5 (4.7–6.4)	–	–	–
**Exercise Habits**		–	–	–
Never	72.8 (71.1–74.5)	–	–	–
Less than once a week	6.4 (5.5–7.5)	–	–	–
Once a week	4.0 (3.2–4.9)	–	–	–
Twice a week	4.0 (3.3–4.7)	–	–	–
Three or more times a week	7.2 (6.3–8.1)	–	–	–
Missing	5.7 (4.9–6.6)	–	–	–
**Mother's socioeconomic status quintile**		–	–	–
1 (poorest)	20.8 (19.6–22.0)	–	–	–
2	20.6 (19.2–22.0)	–	–	–
3	20.2 (18.8–21.7)	–	–	–
4	18.3 (16.7–19.9)	–	–	–
5 (richest)	18.3 (16.9–19.9)	–	–	–
Missing	1.7 (1.3–2.2)	–	–	–
**Maternal overweight**	66.1 (64.4–67.8)	–	–	–
**Maternal obesity**	42.5 (40.1–44.2)	–	–	–

Standard deviation and 95% confidence interval are displayed in parenthesis.

a Both refers to sons and daughters.

### 3.2 Intergenerational transmission of overweight and obesity from mothers to offsprings

**Fig. 2:**
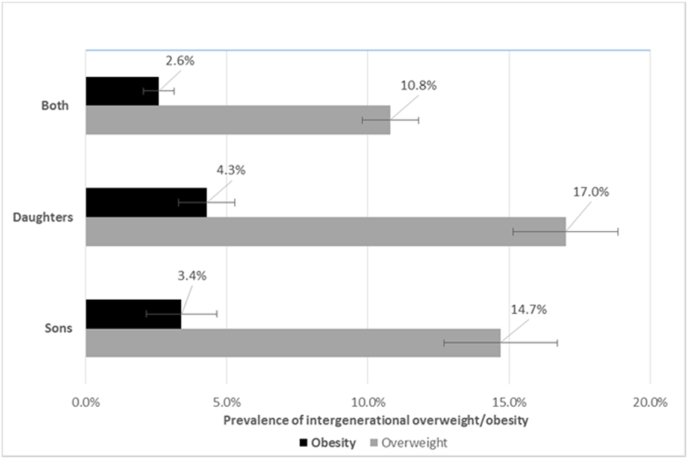
Intergenerational transmission of overweight and obesity from mothers to offsprings stratified by offspring sex, 2017 Note: Error bars represent the 95% confidence intervals. Both refers to sons and daughters.

Combined, the prevalence of intergenerational overweight and obesity was 15.7% (95% CI 14.4%–17.2%) and 3.9% (95% CI 3.1%–4.8%), respectively (Fig. 2). The prevalence of intergenerational overweight, as shown in Fig. 2, was higher for girls (17.2%; 95% CI 15.4%–19.3%) than boys (14.2%; 95% CI 12.3%–16.5%), with overlapping CIs. Similarly, intergenerational obesity was higher for girls (4.4%; 95% CI 3.4%–5.5%) than boys (3.4%; 95% CI 2.3%–4.9%), with overlapping CIs.

**Fig. 3:**
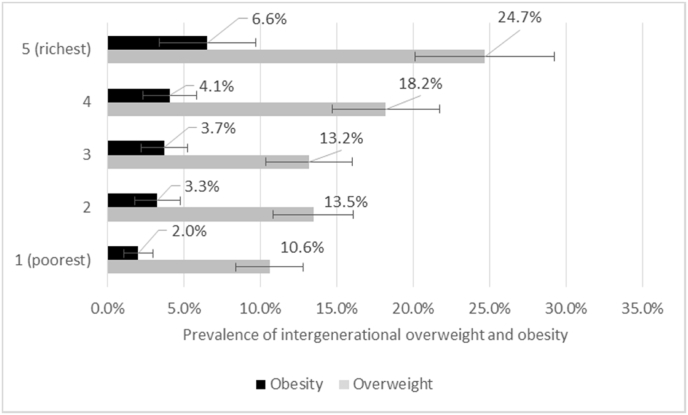
Intergenerational transmission of overweight and obesity from mothers to offsprings stratified by socioeconomic quintile, 2017 Note: Error bars represent the 95% confidence intervals.

As shown in Fig. 3, the prevalence of intergenerational overweight and obesity increased with socioeconomic status quintile. The prevalence of overweight was highest among the wealthiest 20% of mothers (24.7%; 95% CI 20.4%–29.5%) as is the prevalence of obesity (6.6%; 95% CI 4.0%–10.5%).

### 3.3 Inequality in intergenerational overweight and obesity using the concentration index

The concentration indices for intergenerational overweight were positive and statistically significant for both sexes (0.19 and 0.16 for girls and boys, respectively). These indices were also significantly positive for both sexes (0.20 and 0.27, respectively, for girls and boys) for intergenerational obesity. For both boys and girls, the concentration indices for intergenerational overweight and obesity (0.17 and 0.23, respectively) were positive and statistically significant. These results mean that intergenerational transmission of overweight and obesity from mothers to offsprings occurs more often in wealthier than poorer households in South Africa.

### 3.4 Decomposition of socioeconomic inequality in intergenerational overweight and obesity

**Fig. 4:**
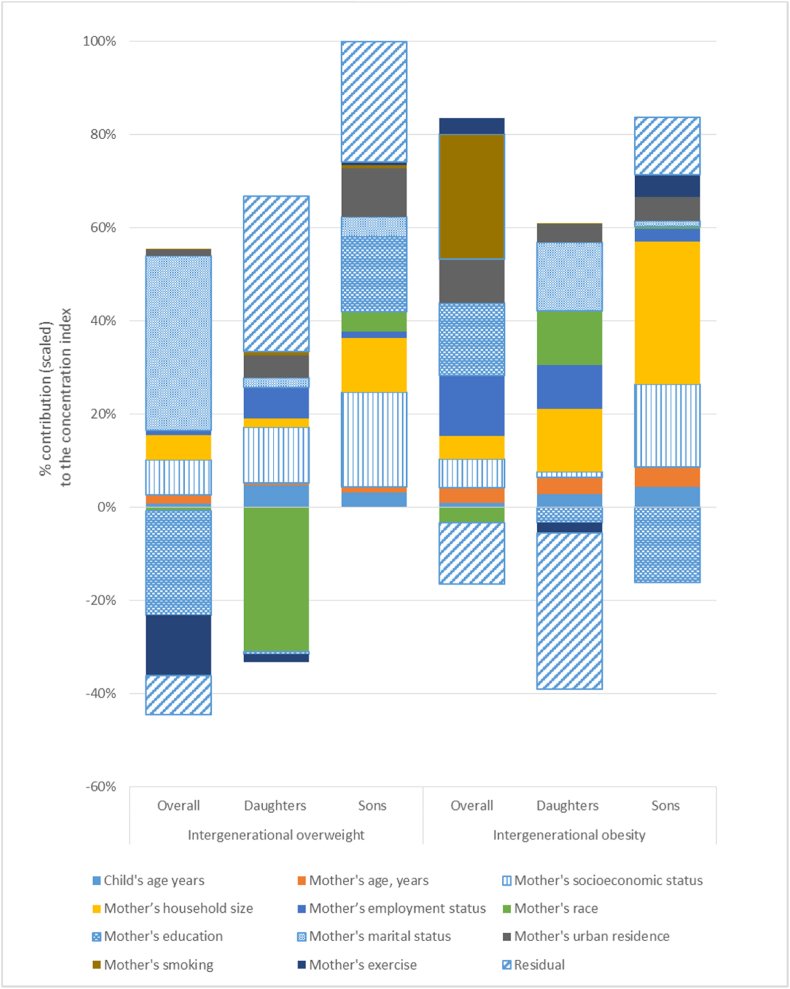
Contribution of determinants to inequality in the intergenerational transmission of overweight and obesity from mothers to offspring in South Africa, 2017 Note: Overall refers to sons and daughters.

Socioeconomic inequality in the intergenerational transmission of overweight from mothers to both girls and boys, as shown in Fig. 4, is attributed mainly to the mother's marital status (+38%), socioeconomic status (+8%) and education (−22%). For girls, a mother's socioeconomic status (+16%), employment status (around +6%) and residing in urban areas (+3%) contribute positively to the socioeconomic inequalities in intergenerational overweight. For boys, mothers' education (+18%), socioeconomic status (+22%) and household size (+13%) contribute positively to the socioeconomic inequalities in intergenerational overweight. Other contributing factors to the socioeconomic inequality in the intergenerational transmission of overweight for boys or girls include a child's age, mother's marital status and race group.

Socioeconomic inequality in the intergenerational transmission of obesity for girls and boys combined is attributed mainly to the mother's smoking status (+25%), education (+13%), employment status (+12%), urban residence (+8%), household size (+6%) and exercise habit (about +4%). For girls, mothers' marital status (+16%), household size (+14%), mothers' race (+12%), mothers' employment status (+8%), urban residence (+6%), and mothers' socioeconomic status (+4%) were positive contributors to socioeconomic inequalities in intergenerational obesity while mothers' marital status (−4%) was a negative contributor. Mothers' household size (+32%), and socioeconomic status (+14%) were positive contributors, while mothers' education was a negative contributor (−18%) to socioeconomic inequality in intergenerational obesity for boys. About +10% of the inequality remained unexplained for intergenerational obesity for boys, with a larger share remaining unexplained for girls. The appendix shows detailed results of the decomposition of socioeconomic inequality in intergenerational overweight and obesity.

## 4 Discussion

This study finds that intergenerational transmission of overweight and obesity occurs more often among the wealthier than poorer mother-offspring pairs (for both girls and boys). The main contributing factors to socioeconomic inequality in intergenerational overweight or obesity differed slightly when the analysis was stratified by offspring sex. For instance, the main contributors to socioeconomic inequality in intergenerational overweight for boys include mothers' education, socioeconomic status and household size. For girls, the factors include mother's socioeconomic status, employment status and urban residence.

It is not surprising that this study demonstrates the presence of intergenerational overweight and obesity, especially among more affluent mother-offspring pairs in South Africa. Evidence shows a significant concentration of overweight and obesity, especially in the adult population, among wealthier individuals in low- and middle-income countries (McLaren, 2007; Shrewsbury & Wardle, 2008; Sobal & Stunkard, 1989), including South Africa (Alaba & Chola, 2014; Cois & Day, 2015; Sartorius et al., 2015) and other countries in sub-Saharan Africa (Abrha et al., 2016; Dake et al., 2011; Letamo, 2011; Olatunbosun et al., 2011; Steyn et al., 2011). This relationship between SES and overweight or obesity is not always linear for high- and low-income countries. For example, while some high-income countries show a positive relationship, lower SES may be associated with obesity for adults and children in many other high-income countries (McLaren, 2007; Strugnell et al., 2020). The finding in South Africa that mother-offspring pairs living with overweight or obesity are more common among wealthier mothers means that overweight or obesity among the rich in South Africa perpetuates itself, irrespective of the offspring's sex.

Certain mothers' socio-demographic factors significantly explained socioeconomic inequality in intergenerational overweight or obesity in South Africa. For example, socioeconomic status is a major contributor to the intergenerational transmission of overweight and obesity for both girls and boys. This finding agrees with Balasooriya and colleagues' finding that parental socioeconomic status significantly contributes to BMI and overweight in adult children (Balasooriya et al., 2021), resulting from differences in consumption patterns between the rich and the poor. Studies among adults from sub-Saharan Africa have also shown a positive relationship between educational attainment and obesity (Adeboye et al., 2012; Dalal et al., 2011; Letamo, 2011; Mfenyana et al., 2006). Interestingly, as found in this paper, apart from inequalities in intergenerational obesity for boys, mothers' education positively contributed to the socioeconomic inequality in intergenerational overweight and obesity for girls and boys. Differences in food consumption patterns and sedentary lifestyles, which may be explained by education, could explain this finding, as discussed later on. Household size explained socioeconomic inequality in intergenerational obesity for girls and boys. Because household size increases faster among poorer than wealthier households, it was found that household size increases inequalities in intergenerational obesity among wealthier mother-offspring pairs than poorer pairs. This is corroborated by a study from Mexico showing that increased household size decreases the odds of overweight and obesity in mother-offspring pairs (Cauich-Viñas et al., 2019). Consistent with a previous study from Australia (Balasooriya et al., 2021), our study found that mothers’ employment status explained socioeconomic inequality in intergenerational obesity for girls.

In the literature, racial disparities have been reported for obesity and overweight (Averett et al., 2014). Similarly, in our study, the self-reported race was found to contribute positively to the socioeconomic inequality in the intergenerational transmission of obesity from mothers to girls. While it remains unclear how race may contribute to perpetuating overweight and obesity between generations, an earlier qualitative study provided insight into the intergenerational dynamics, complexity, and relationship with food and exercise among Black African mother and daughter pairs in an urban setting in South Africa (Phillips et al., 2016). That study found that mothers living with obesity and daughters with normal weight shared comparable perceptions of healthy food and exercise rooted in their daily life (Phillips et al., 2016). Still, these mothers tended to report healthier eating and exercising than their daughters, which was linked to ageing or ill-health (Phillips et al., 2016). While this is complex, daughters may have assimilated views of healthy eating and exercise in childhood or adolescence through continuous exposure; thereby maintaining a normal weight (Phillips et al., 2016). Although not directly linked to race, Classen noted that while the intergenerational transmission of BMI or obesity may be complex, culture and family values might influence the transmission of BMI and obesity from parents to offsprings (Classen, 2010).

Mother's marital status positively contributed to the socioeconomic inequality in intergenerational overweight for girls. Although the explanations for the contribution of marital status to the intergenerational transmission of overweight and obesity is not well understood, it is purported that marriage, as a significant life transition, may alter the eating habits of individuals. The changes in eating habits may result in excessive weight gain, possibly due to shifts in roles and responsibilities after getting married within the African context (Faber & Kruger, 2005; Okop et al., 2015; Puoane et al., 2002). Moreover, parents and children sharing common environmental factors, including similar food consumption choices, could partly account for the observed intergenerational transmission of obesity from mothers to girls (Classen & Thompson, 2016). There is a need for more studies to unpack the causal pathways through which marital status affects intergenerational overweight and obesity.

Living in urban areas contributes to socioeconomic inequality in intergenerational obesity for girls and boys. This may result from differences in available diets between urban and rural populations, processed food consumption and lifestyles, including increased physical inactivity and sedentary behaviour characteristic of urban populations (Pisa & Pisa, 2017).

The finding that mothers' exercise habit contributes to the socioeconomic inequality in intergenerational obesity for girls and boys is expected as parents and offsprings share inactive or sedentary lifestyle behaviours (e.g., reduced physical activity and increased sedentary behaviours) (Classen & Thompson, 2016). However, it is unclear why exercise habit was not a significant determining factor for socioeconomic inequalities in intergenerational overweight. While this may be speculative, it may point toward the complexity in the underlying transition from overweight to obesity that cannot be fully uncovered using the quantitative analysis in this paper. Factors such as neighbourhood safety influence individuals’ physical activity levels (Malambo et al., 2017; Oyeyemi et al., 2012) and could explain why living in urban areas contributes to socioeconomic inequality in intergenerational overweight and obesity in South Africa. Lokuruka (2013) argued that the habit of sitting and watching television for prolonged periods, and the long time children spend playing video games in wealthy and middle-class urban dwellers, may contribute to the rise of overweight and obesity in parents and children in wealthier households in sub-Saharan Africa.

The finding that mothers’ smoking, which is unequally distributed between wealthier and poorer women in South Africa, contributes to the socioeconomic inequality in intergenerational obesity for girls and boys is also expected as lifestyle behaviours, including smoking, play a part in the intergenerational cycle of overweight and obesity (World Health Organization, 2007).

A significant portion of socioeconomic inequality in intergenerational obesity especially, could not be explained by the factors included in our model. This finding is not surprising as “the transmission of health outcomes between generations is a complicated process governed by a myriad of factors including genetics, culture, family values and consumption choice” (Classen, 2010, p. 32), some of which are not quantifiable or included in existing datasets. Therefore, unobserved and unquantifiable factors such as individuals’ circumstances, behaviours, genetic factors, family values, and culture (Balasooriya et al., 2021; Classen, 2010) may be responsible for sizeable unexplained factors in our analysis. The transition from overweight to obesity in South Africa may be attributed to significant changes in unexplained or unquantifiable factors, showing why unexplained factors are more pronounced for socioeconomic inequalities in intergenerational obesity than for intergenerational overweight. Our analysis observed that while the prevalence of overweight was higher among girls, the prevalence of obesity was slightly lower among girls, highlighting the possible complexity in this transition between overweight and obesity. Clearly, this area of research is important and relevant for future investigation to unpack some of the complexities and understand the mechanisms for the transition from overweight to obesity for girls and boys and why the determining factors are different for mother-daughter and mother-son pairs. The differences in factors explaining socioeconomic inequality in the intergenerational transmission of overweight and obesity by offspring sex are difficult to provide convincing arguments using the quantitative analysis in this paper without understanding the complexities in the transmission mechanisms, including the impact of the many unexplained factors. We, therefore, note that qualitative assessments could uncover these nuances and better explain the socioeconomic inequalities in intergenerational overweight and obesity in the country.

The paper's findings have implications for policy and further research. There is a need for more research assessing the relationships between the intergenerational transmission of overweight/obesity and socioeconomic status and how this may differ for mother-son and mother-daughter pairs. This will help us understand the pathways and mechanisms underlying this transmission to reduce South Africa's health inequalities (Ataguba et al., 2011). There is a need for nuanced exploration in future studies, possibly through rigorous qualitative studies, on the pre-conception of intergenerational transmission of overweight or obesity and identifying the social and environmental determinants promoting the onset of childhood overweight and obesity over time after birth. The significant intergenerational transmission of obesity in South Africa requires policy and interventions to focus on reducing excessive weight gain in women of childbearing age. This is crucial for preventing premature death and disease, reducing the economic burdens and strains on social protection systems and contributing to attaining the country's national development goals. Specifically, as socioeconomic inequality was to the disadvantage of the rich, care is needed as interventions and resources targeting wealthier women to reduce intergenerational overweight or obesity could instead exacerbate other health inequalities. Therefore, interventions should target women across all SES groups in South Africa, focusing especially on addressing the social determining factors, many of which lie outside the health sector, which explain significant intergenerational transmission of overweight and obesity. As an entry point, family-based interventions are examples of cost-effective strategies to reduce obesity with weight loss in children, usually accompanied by parental weight loss (Boutelle et al., 2012; Wrotniak et al., 2004).

This study has some limitations. Some of the factors that explain socioeconomic inequalities in intergenerational overweight and obesity were not captured in this study, mainly because they may be intangible or not contained in the NIDS dataset. Cultural factors, for instance, were not included in our analysis apart from the use of race categories. Also, factors such as dietary lifestyle and breastfeeding practices were not available in the NIDS dataset but could explain socioeconomic inequalities in intergenerational overweight and obesity**.** In addition, there is missing data for resident fathers. Also, this paper assumes intergenerational transmission of overweight and obesity as simply observing overweight or obesity in a mother-offspring pair. While this may be so in most cases and will not change our findings qualitatively, an offspring's overweight or obesity status may be unrelated to the mother's. Also, using a dummy variable indicating obesity or overweight in a mother-child pair treats all obesity and overweight as the same, ignoring the weight spectrum and misses the “intensity” of the intergenerational obesity or overweight. Future research may be needed to examine whether the conclusions in this paper will change after accounting for this “intensity”. Relatedly, intertemporal analysis using datasets for two time periods (preferably with a ten-year gap) may better explain intergenerational obesity. However, doing this with the current longitudinal data was limiting as the sample size for mother-child pairs reduces substantially over time, among other things. Also, this paper's self-reported data for physical exercise may be prone to bias. However, most household surveys globally rely on self-reports for physical activity, and our results are consistent with others in the literature as discussed in the paper. The standard concentration index was used to quantify socioeconomic inequality in intergenerational overweight and obesity instead of the Wagstaff's and Erreygers' normalisation, which may provide counter-intuitive results for policy (Ataguba, 2022). However, future research could explore epidemiological approaches to identifying the factors associated with intergenerational overweight and obesity. These limitations notwithstanding, the study has some strengths, including using objective measures and contemporaneous observations of measured BMI for mothers and BMI-for-age and weight-for-height z-scores for offsprings.

## 5 Conclusions

Intergenerational transmission of overweight and obesity has been reported in the literature, with a complex pathway. However, there is a dearth of studies assessing socioeconomic inequalities in intergenerational overweight and obesity, especially in South Africa, with one of the highest burdens of obesity in sub-Saharan Africa. The need to secure future generations and reduce the current levels of health inequalities in South Africa demands an understanding of the nature of intergenerational inequalities, including the transmission of overweight and obesity between mothers and offsprings. Besides, policies are needed to address the factors that underlie this transmission, as identified in this study. We argue that the critical social determinants of health inequalities identified in this paper for intergenerational overweight and obesity provide policy insights to reduce health inequalities in South Africa's future generations.

## Ethical statement

Although the data were publicly available, this study received ethics approval from the Human Research Ethics Committee at the University of Cape Town (HREC Reference 409/2019).

## Author statement

Mweete Debra Nglazi carried out the statistical analysis with input from John Ele-Ojo Ataguba and wrote the first draft of the manuscript. All authors conceptualised the research, reviewed the results, revised the manuscript, read and approved the final version of the manuscript submitted for publication.

## Funding

The work reported herein was made possible through funding by the 10.13039/501100001322South African Medical Research Council (SAMRC) through its 10.13039/501100015777Division of Research Capacity Development under the National Health Scholarship Programme from funding received from the Public Health Enhancement Fund/South African 10.13039/100009041National Department of Health. The content hereof is the sole responsibility of the authors and does not necessarily represent the official views of the SAMRC. JEA is supported by the Canada Research Chair. The funder had no role in the design of the study and collection, analysis, and interpretation of data and in writing the manuscript.

## Declaration of competing interest

None.
