# Supplementary material for: Socioeconomic inequalities in intergenerational overweight and obesity transmission from mothers to offsprings in South Africa
Source: SSM Popul Health. 2022 Aug 6;19:101170. doi: 10.1016/j.ssmph.2022.101170 (PMC9399383; doi:10.1016/j.ssmph.2022.101170)
Supplement: Multimedia component 1 [file mmc1.docx]

# Appendix

**Table S1: Decomposition of concentration index for intergenerational transmission of overweight from mothers to offspring in South Afica, 2017**

|  | Sons | | | Daughters | | | Both§ | | |
| --- | --- | --- | --- | --- | --- | --- | --- | --- | --- |
|  | Concentration indices | Elasticities | Contributions | Concentration indices | Elasticities | Contributions | Concentration indices | Elasticities | Contributions |
| Child's age years | 0.013  (0.010) | 0.352  (0.382) | 0.005  (0.007) | 0.037**  (0.003) | 0.717  (230.362) | 0.026  (8.349) | 0.025**  0.007) | 0.529  (2.525) | 0.013  (0.062) |
| Mother's age, years | 0.013**  (0.003) | 0.182  (0.910) | 0.002  (0.012) | 0.020**  (0.003) (0.003) | 0.134  (29.266) | 0.003  (0.563) | 0.016**  (0.002) | 1.719  (7.240) | 0.028  (0.116) |
| Mother's socioeconomic status | 0.632**  (0.018) | 0.052  (0.068) | 0.033  (0.042 | 0.628**  (0.015) | 0.106  (182.103) | 0.066  (114.786) | 0.631**  (0.011) | 0.191  (0.763) | 0.121  (0.481) |
| Household size | -0.168**  (0.008) | -0.113  (0.770) | 0.019  (0.128) | -0.171**  (0.007) | -0.060  (3.436) | 0.010  (0.589 | -0.170**  (0.005) | -0.487  (6.642) | 0.083  (1.121) |
| **Mother’s employment status** |  |  |  |  |  |  |  |  |  |
| Unemployed | Reference | Reference | Reference | Reference | Reference | Reference | Reference | Reference | Reference |
| Employed | 0.155**  (0.010) | 0.014  (0.134) | 0.002  (0.021) | 0.172**  (0.011) | 0.214  (10.347) | 0.037  (1.770) | 0.163**  (0.008) | 0.098  (1.252) | 0.016  (0.208) |
| **Mother's population group** |  |  |  |  |  |  |  |  |  |
| Black African | Reference | Reference | Reference | Reference | Reference | Reference | Reference | Reference | Reference |
| Coloured | 0.129**  (0.055) | 0.007  (0.052) | 0.001  (0.006) | 0.197**  (0.050) | 0.010  (0.369) | 0.002  (0.075) | 0.162**  (0.036) | 0.010  (0.019) | 0.002  (0.003) |
| Asian/Indian | 0.588**  (0.100) | 0.003  (0.010) | 0.002  (0.006) | 0.592**  (0.065) | 0.001  (0.005) | 0.001  (0.003) | 0.589**  (0.059) | 0.002  (0.005) | 0.001  (0.003) |
| White | 0.805**  (0.050) | 0.005  (0.018) | 0.004  (0.014) | 0.854**  (0.041) | -0.204  (499.825) | -0.174  (425.693) | 0.829**  (0.032) | -0.016  (1.224) | -0.014  (1.017) |
| **Mother's education** |  |  |  |  |  |  |  |  |  |
| No schooling | Reference | Reference | Reference | Reference | Reference | Reference | Reference | Reference | Reference |
| Primary | -0.525**  (0.031) | -0.008  (0.488) | 0.004  (0.255) | -0.441**  (0.038) | 0.054  (15.145) | -0.024  (6.713) | -0.485**  (0.024) | 0.021  (6.419) | -0.010  (3.130) |
| Secondary | 0.298**  (0.023) | 0.189  (0.393) | -0.012  (0.025) | -0.082**  (0.011) | 0.732  (30.720) | -0.060  (2.424) | -0.073**  (0.009) | 5.467  (24.511) | -0.401  (1.798) |
| Tertiary | 0.210  (0.023) | 0.111  (0.129) | 0.033  (0.039) | 0.355**  (0.022) | 0.224  (327.114 | 0.080  (112.199) | 0.325**  (0.016) | 0.169**  (0.060) | 0.055**  (0.020) |
| Vocational | 0.210  0.232) | 0.002  (0.009) | 0.000  (0.005) | 0.307**  (0.115) | 0.003  (0.108) | 0.001  (0.033) | 0.260**  (0.118) | 0.002  (0.003) | 0.001  (0.001) |
| **Mother's marital status** |  |  |  |  |  |  |  |  |  |
| Married | Reference | Reference | Reference | Reference | Reference | Reference | Reference | Reference | Reference |
| Living with partner | 0.040  (0.043) | -0.090  (0.735) | -0.004  (0.028) | 0.05  (0.046) | -0.114  (19.542) | -0.006  (1.372) | 0.045  (0.032) | -0.523  (13.632) | -0.024  (0.560) |
| Widow | -0.157*  (0.082) | 0.001  (0.009) | -0.000  (0.001) | -0.241**  (0.101) | -0.002  (1.270) | 0.000  (0.299) | -0.196**  (0.064) | -3.043  (3.935) | 0.598  (0.844) |
| Divorced or separated | 0.359**  (0.083) | -0.008  (0.046) | -0.003  (0.017) | 0.447**  (0.075) | -0.006  (108.909) | -0.003  (50.644) | 0.408**  (0.057) | -0.005  (0.029) | -0.002  (0.012) |
| Never married | -0.132**  (0.016) | -0.106  (0.606) | 0.014  (0.081) | 0.160**  (0.015) | -0.128  (6.868) | 0.020  (1.111) | -0.145**  (0.011) | -0.143  (10.133) | 0.021  (1.490) |
| **Area of residence** |  |  |  |  |  |  |  |  |  |
| Rural | Reference | Reference | Reference | Reference | Reference | Reference | Reference | Reference | Reference |
| Urban | 0.201**  (0.011) | 0.083  (0.153) | 0.017  (0.031) | 0.202**  (0.012) | 0.128  (77.466) | 0.026  (15.721) | 0.202**  0.008) | 0.099  (4.924) | 0.020  (1.010) |
| **Mother smoking** |  |  |  |  |  |  |  |  |  |
| Not smoking | Reference | Reference | Reference | Reference | Reference | Reference | Reference | Reference | Reference |
| Smoking | 0.167**  (0.058) | 0.008  (0.058) | 0.001  (0.009) | 0.284**  (0.062) | 0.016  (0.283) | 0.005  (0.078) | 0.225**  (0.044) | 0.014  (0.364) | 0.003  (0.080) |
| **Exercise Habits** |  |  |  |  |  |  |  |  |  |
| Never | Reference | Reference | Reference | Reference | Reference | Reference | Reference | Reference | Reference |
| Less than once time a week | 0.130**  (0.063) | 0.001  (0.032) | 0.000  (0.004) | 0.167**  (0.058) | -0.000  (0.182) | -0.000  (0.032) | 0.149**  (0.044) | 0.001  (0.031) | 0.000  (0.004) |
| Once a week | 0.347**  (0.079) | 0.019  (0.012) | 0.006  (0.005) | -0.055  (0.083) | 0.001  (0.048) | -0.000  (0.003) | 0.196**  (0.071) | 0.010  (0.009) | 0.002  (0.002) |
| Twice a week | 0.122  (0.077) | -0.006  (0.136) | -0.001  (0.015) | 0.263**  (0.071) | -0.025  (4.414) | -0.007  (1.093) | 0.193**  (0.054) | -0.008  (1.493) | -0.001  0.281) |
| Three or more times a week | 0.228**  (0.059) | -0.021  (0.286) | -0.005  (0.061) | 0.295**  (0.050) | -0.009  (29.105) | -0.003  (8.549) | 0.264**  (0.039) | -0.775  (2.124) | -0.204  (0.524) |
| Residual |  |  | 0.042  (0.385) |  |  | 0.184  (233.701) |  |  | -0.133  (2.131) |
| Total |  |  | 0.161**  (0.044) |  |  | 0.185**  (0.031) |  |  | 0.172**  (0.026) |

Significance levels are denoted as follows: ** p< 0.05, *p< 0.10. Standard error displayed in parentheses.

§Both refers to sons and daughters.

**Table S2** **Decomposition of concentration index for intergenerational transmission of obesity from mothers to offsping in South Afica, 2017**

|  | Sons | | | Daughters | | | Both§ | | |
| --- | --- | --- | --- | --- | --- | --- | --- | --- | --- |
|  | Concentration indices | Elasticities | Contributions | Concentration indices | Elasticities | Contributions | Concentration indices | Elasticities | Contributions |
| Child's age years | 0.013  (0.010) | 1.442  (33.521) | 0.019  (0.547) | 0.034**  0.009) | 0.733  (98.758) | 0.025  (3.199) | 0.025**  (0.007) | 0.115  (110.932) | 0.003  (2.966) |
| Mother's age, years | 0.013**  0.003) | -0.293  30.425) | -0.004  (0.436 | 0.019**  (0.003) | 1.731  (152.010) | 0.032  (2.707) | 0.016**  (0.002) | 0.700  (131.497) | 0.011  (2.065) |
| Mother's socioeconomic status | 0.632**  (0.018) | 0.123  (3.133) | 0.078  (1.977) | 0.629**  (0.015) | 0.016  (2.519) | 0.010  (1.585) | 0.606**  (0.012) | 0.034  (4.636) | 0.021  (2.818) |
| Household size | -0.168**  (0.008) | -0.799  (56.900) | 0.134  (9.674) | -0.169**  (0.006) | -0.711  (56.740) | 0.120  (9.623) | -0.170**  (0.005) | -0.092  (83.074) | 0.016  (14.139) |
| **Mother’s employment status** |  |  |  |  |  |  |  |  |  |
| Unemployed | Reference | Reference | Reference | Reference | Reference | Reference | Reference | Reference | Reference |
| **Employed** | 0.155**  (0.010) | 0.078  (2.444) | 0.012  (0.378) | 0.174**  (0.010) | 0.486  (40.116) | 0.084  (6.861) | -0.307**  (0.016) | -0.140  (10.723) | 0.043  (3.271) |
| **Mother's population group** |  |  |  |  |  |  |  |  |  |
| Black African | Reference | Reference | Reference | Reference | Reference | Reference | Reference | Reference | Reference |
| Coloured | 0.129**  0.055) | -0.093  (12.083) | -0.012  1.475) | 0.206**  (0.048) | 0.090  (6.234) | 0.018  (1.347) | 0.162**  (0.036) | -0.013  (52.992) | -0.002  (8.035) |
| Asian/Indian | 0.588**  0.100) | 0.006  (0.336) | 0.003  (0.185) | 0.608**  (0.058) | -0.001  (1.178) | -0.001  (0.734) | 0.589**  0.059) | 0.004  (0.276) | 0.003  (0.156) |
| White | 0.805**  (0.050) | 0.013  (0.452) | 0.011  (0.361) | 0.859**  (0.035) | 0.100  (5.629) | 0.086  (4.824) | 0.829**  (0.032) | -0.013  (1.866) | -0.011  (1.550) |
| **Mother's education** |  |  |  |  |  |  |  |  |  |
| No schooling | Reference | Reference | Reference | Reference | Reference | Reference | Reference | Reference | Reference |
| Primary | -0.525**  (0.031) | 0.174  (3.210) | -0.092  (1.742 | -0.438**  (0.037) | 0.113  (53.865) | -0.050  (23.566) | -0.485**  (0.024) | -0.111  (3.226) | 0.054  (1.589) |
| Secondary | -0.064**  (0.014) | 0.522  80.384) | -0.033  (5.028) | -0.081**  (0.011) | 0.540  (71.298) | -0.044  (5.739) | -0.073**  (0.009) | 0.734  (91.872) | -0.054  (6.340 |
| Tertiary | 0.298**  (0.023) | 0.180  (11.817) | 0.054  (3.501) | 0.343**  (0.021) | 0.182  (20.514) | 0.063  (6.889) | 0.325**  (0.016) | 0.149  (3.420) | 0.049  (1.107) |
| Vocational | 0.210  (0.232) | 0.001  (0.322) | 0.000  (0.073) | 0.291**  (0.111) | 0.004  (0.291) | 0.001  (0.093) | 0.260**  (0.118) | 0.008  (0.114) | 0.002  (0.033) |
| **Mother's marital status** |  |  |  |  |  |  |  |  |  |
| Married | Reference | Reference | Reference | Reference | Reference | Reference | Reference | Reference | Reference |
| Living with partner | 0.040  (0.043) | 0.044  (7.886) | 0.002  (0.349) | 0.062  (0.045) | -0.086  (39.732) | -0.005  2.992) | 0.045  (0.032) | -0.589  (294.381) | -0.027  (13.291) |
| Widow | -0.157*  (0.082) | -0.003  (0.607) | 0.001  (0.093) | 0.256**  (0.092) | 0.274  (26.782) | -0.070  (6.612) | -0.196**  (0.064) | -0.024  (1.126) | 0.005  (0.220) |
| Divorced or separated | 0.359**  (0.083) | -0.004  (0.951) | -0.001  (0.333) | 0.463**  (0.071) | -0.043  (6.857) | -0.020  (3.215) | 0.408**  (0.057) | -0.008  (8.532) | -0.003  (3.368) |
| Never married | -0.132**  (0.016) | -0.029  (14.117) | 0.004  (1.815) | -0.166**  (0.015) | -1.365  (145.950) | 0.227  (24.405) | -0.145**  (0.011) | -0.196  (28.046) | 0.029  (4.110) |
| **Area of residence** |  |  |  |  |  |  |  |  |  |
| Rural | Reference | Reference | Reference | Reference | Reference | Reference | Reference | Reference | Reference |
| Urban | 0.201**  (0.011) | 0.112  (5.467) | 0.023  (1.103) | 0.201**  (0.011) | 0.169  (17.540) | 0.034  (3.558) | 0.202**  (0.008) | 0.154  (8.177) | 0.031  (1.627) |
| **Mother smoking** |  |  |  |  |  |  |  |  |  |
| Not smoking | Reference | Reference | Reference | Reference | Reference | Reference | Reference | Reference | Reference |
| Smoking | 0.167**  (0.058) | -0.002  (3.873) | -0.000  (0.647) | 0.317**  (0.060) | 0.006  (1.123) | 0.002  (0.365) | 0.225**  (0.044) | 0.391  (13.037) | 0.088  (2.946) |
| **Exercise Habits** |  |  |  |  |  |  |  |  |  |
| Never | Reference | Reference | Reference | Reference | Reference | Reference | Reference | Reference | Reference |
| Less than once time a week | 0.130**  (0.063) | 0.045  (0.156) | 0.006  (0.020) | 0.158**  (0.056) | -0.020  (25.329) | -0.003  (3.224) | 0.149**  (0.044) | 0.042  (1.343) | 0.006  (0.198) |
| Once a week | 0.347**  (0.079) | 0.036  (0.404) | 0.013  (0.132) | -0.019  (0.082) | 0.005  (0.594) | -0.000  (0.026) | 0.196**  (0.071) | 0.025  (0.064) | 0.005  (0.012) |
| Twice a week | 0.122  (0.077) | 0.004  (0.464) | 0.001  (0.058) | 0.202**  (0.075) | 0.077  (3.330) | 0.016  (0.720) | 0.193**  (0.054) | 0.028  (1.877) | 0.005  (0.369) |
| Three or more times a week | 0.228**  0.059) | 0.009  (0.432) | 0.002  (0.099) | 0.277**  (0.048) | -0.118  (10.137) | -0.033  (2.543) | 0.264**  (0.039) | -0.022  (1.031) | -0.006  (0.274) |
| Residual |  |  | 0.054  (8.718) |  |  | -0.298  (35.498) |  |  | -0.043  (13.573) |
| Total |  |  | 0.273**  (0.121) |  |  | 0.196**  (0.058) |  |  | 0.225**  (0.064) |

Significance levels are denoted as follows: ** p< 0.05, *p< 0.10. Standard error displayed in parentheses.

§Both refers to sons and daughters.
